# Supplementary material for: Interplay of the disorder and strain in gallium oxide
Source: Sci Rep. 2022 Sep 13;12:15366. doi: 10.1038/s41598-022-19191-8 (PMC9470558; doi:10.1038/s41598-022-19191-8)
Supplement: Supplementary file 1 — Supplementary Information. [file 41598_2022_19191_MOESM1_ESM.pdf]

Supplementary material for

Interplay of the disorder and strain in gallium oxide

Alexander Azarov[1,a], Vishnukanthan Venkatachalapathy[1,2], Platon Karaseov[3], Andrei Titov[3], Konstantin Karabeshkin[3], Andrei Struchkov[3], and Andrej Kuznetsov[1,b]

[1] *Department of Physics, Centre for Materials Science and Nanotechnology, University of Oslo, PO Box 1048 Blindern, N-0316 Oslo, Norway*

[2] *Department of Materials Science, National Research Nuclear University, "MEPhI", 31 Kashirskoe Hwy, 115409 Moscow, Russian Federation*

[3] *Peter the Great St.-Petersburg Polytechnic University, St.-Petersburg, Russia*

[a] alexander.azarov@smn.uio.no
[b] andrej.kuznetsov@fys.uio.no

Fig. S1 illustrates a difference in the volumetric distribution of primary defects within one individual collision cascade generated by the 100 keV relatively light ion (P) and low energy (36 keV) medium mass ion (Ni) as calculated by the SRIM code [S1] simulations. It is seen that P ion produces diluted collision cascade consisting of several well defined subcascades generally described by a fractal analysis [S2]. In its turn, collision cascade generated by Ni ion is denser and more compact including the subcascades.

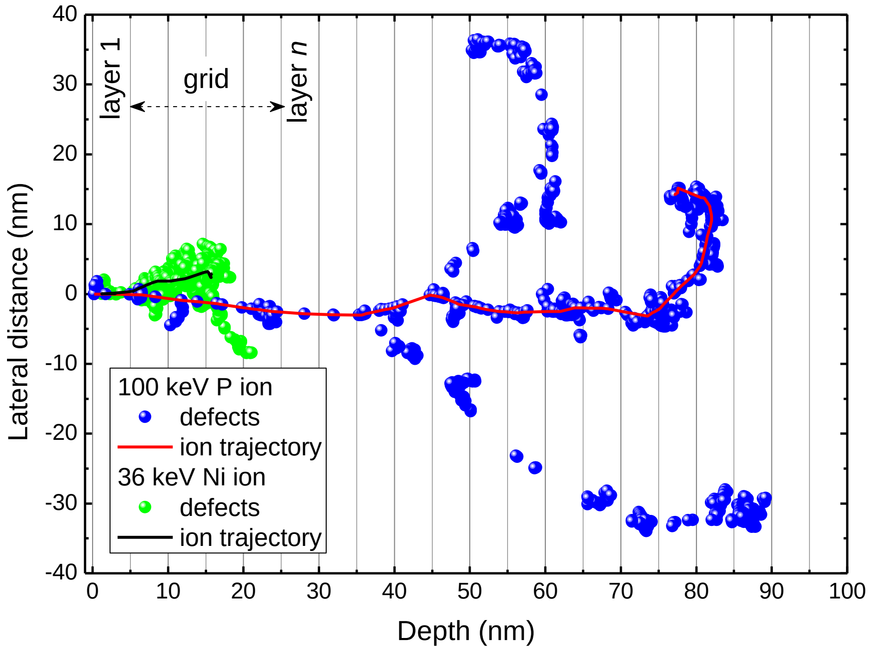

Fig. S1 Projections of individual collision cascades produced by 100 keV P and 36 keV Ni ions in β-Ga_2O_3 as calculated by the SRIM code [S1] simulations.

The details of the algorithm used in the present study to calculate average parameters of the collision cascades can be found elsewhere [S3,S4]. Specifically, for all ions studied the whole implanted region was interpreted as a grid with a 5 nm step, as shown in Fig. S1. Then, collision cascade density was calculated in each grid layer taking into account the subcascade formation. In order to separate defects into subcascades, the distance between the defects belonging to one subcascade was assumed to be ≤2 nm. For simplicity, the subcascades containing <4 defects were excluded from the consideration. Cascade volume and number of defects were calculated for each grid layer. After that the results were averaged over a big number of the individual collision cascades. Importantly, each collision cascade of the cluster ion was constructed by randomly chosen individual cascades produced by the corresponding atoms comprising the cluster.

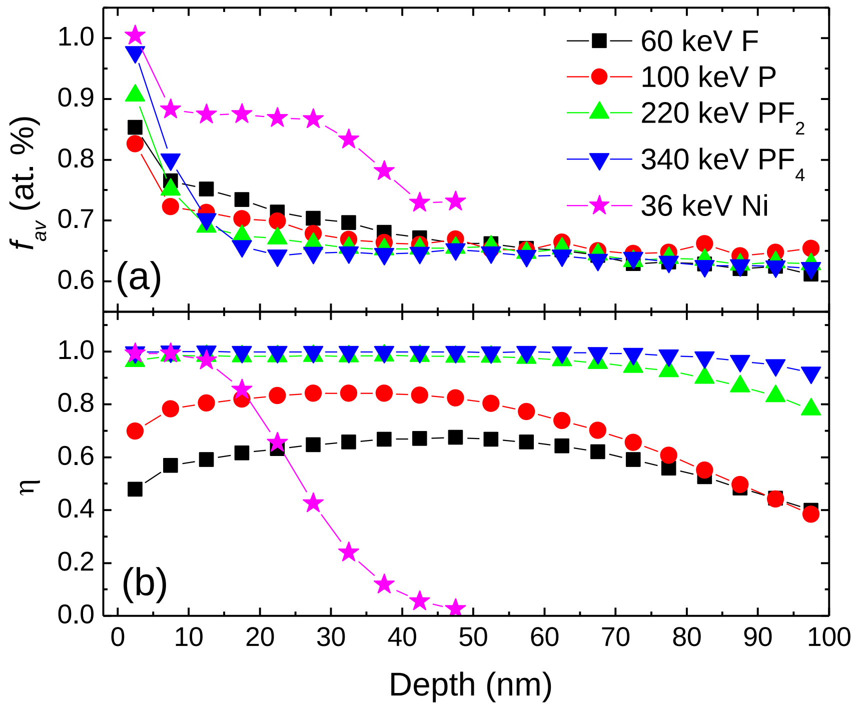

Fig. S2 (a) the average density of collision cascade (f_{av}) and (b) a probability of formation at least one subcascade in the layer under consideration (η) as a function of depth for atomic (Ni, F and P) and cluster (PF_2 and PF_4) ions.

The results of the cascade density calculations are summarized in Fig. S2. It is clearly seen that for cluster ions the average density of the collision cascade increases near the surface region (in the first ~10 nm from the surface); however, with increasing depth it becomes equal to those of the atomic ions., For cluster ions collision cascade density enhancement occurs near the surface due to an efficient overlapping of the cascades generated by the atoms comprising the cluster ion, i.e. F and P ions. At the same time, the probability of subcascade formation (η) is practically equal to unity for cluster ions in the depth range up to R_{pd} (~46 nm for F and P ions), while it is decreases with lowering atomic mass of the ions and η does not exceed 0.6 for F ions in the whole depth range. Fig. S2 shows also that low energy Ni ions produce relatively dense

collision cascade comparable with that of cluster ions near the surface. However, η decreases fast for the depth exceeding R_{pd} of Ni ions (~10 nm).

In order to rationalize the estimations of the collision cascade density and efficiently compare the data for different ions, we have calculated the effective collision cascade density determined as an average collision cascade density multiplied by a subcascade probability in each grid layer i.e. $f_{eff} = f_{av} \times \eta$, resulted in the data in Fig. 3 in the main text.

Finally, it should be mentioned that the low energy Ni ions produce collision cascades with even higher density near the surface region as compared to those for cluster ions (see Fig. S2). However, the surface disorder in the Ni implanted samples is low and comparable with that produced by atomic ions (Fig. 5(a)) for the same DPA value. This effect may be attributed to the proximity of the surface for these implants and, therefore, enhanced defect annihilation at the surface for low energy Ni ions.

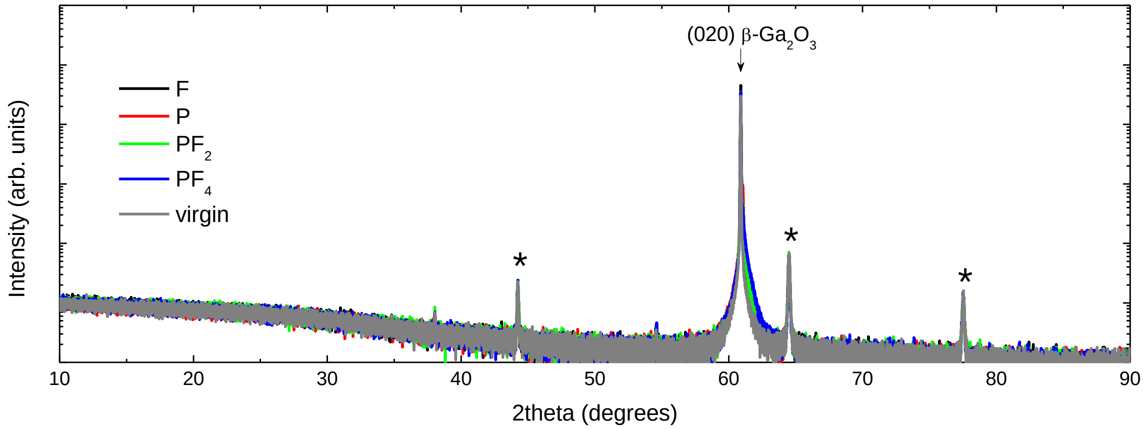

Fig. S3 XRD 2theta scans for the full angular range of the (010) oriented β-Ga_2O_3 samples. The data are shown for the virgin, F, P and PF_n implanted samples as indicated in the legend. Notably, several minor peaks labeled with asterisks are related to the sample holder.

References:

S1. J. F. Ziegler, M. D. Ziegler, and J. P. Biersack, "SRIM—the stopping and range of ions in matter (2010)", Nucl. Instrum. Methods Phys. Res. B **268,** 1818 **(**2010).

S2. K. B. Winterbon, H. M. Urbassek, P. Sigmund, and A. Grasmarti, "Collision Cascades as Fractals" Phys. Scr. **36,** 689 (1987).

S3. P. A. Karaseov, A. Yu. Azarov, A. I. Titov, and S. O. Kucheyev, "Density of Displacement Cascades for Cluster Ions: An Algorithm of Calculation and the Influence on Damage Formation in ZnO and GaN", Semiconductors **43**, 691 (2009).

S4. S. O. Kucheyev, A. Yu. Azarov, A. I Titov, P. A Karaseov, T. M. Kuchumova, "Energy spike effects in ion-bombarded GaN", J. Phys. D: Appl. Phys. **42,** 085309 (2009).
